# A Case Report of Progressive Brown Syndrome?

**DOI:** 10.22599/bioj.110

**Published:** 2018-05-10

**Authors:** A. Coughlan, G. E. Arblaster, J. P. Burke

**Affiliations:** 1 Academic Unit of Ophthalmology and Orthoptics, University of Sheffield, GB; 2 Ophthalmology Department, Royal Hallamshire Hospital, Sheffield, GB

**Keywords:** Brown syndrome, progressive, strabismus surgery

## Abstract

**Aim:**

To report an unusual case of progressive Brown syndrome and the successful surgical treatment.

**Methods:**

A 42-year-old male with a documented 14-year history of progressive Brown syndrome is presented. To improve diplopia symptoms an ipsilateral superior oblique (SO) 7 mm silicone tendon spacer and a contralateral 3 mm superior rectus (SR) recession were performed.

**Results:**

The surgical procedure was successful in reducing the primary position deviation (preoperative 30ΔLHoT 7ΔLXT; one month post surgery 3ΔLHo; one year post surgery 7-8ΔLHo 1-2ΔE) and eliminating the abnormal head posture (AHP). Diplopia was only reported on elevation and depression following surgery.

**Conclusion:**

This case of Brown syndrome is unusual as it was progressive and had a documented history over a number of years prior to surgery. Surgical treatment of Brown syndrome is rarely required, but in this case was successful in improving the patient’s diplopia and AHP.

## Introduction

Brown syndrome is an ocular motility defect where the affected eye(s) does not elevate in adduction but has full depression in adduction (Ansons and Davis 2014). The limitation is the same on versions, ductions and passive rotations (positive forced duction test (FDT)) (Manley and Rizwan 2011). Brown syndrome can be classified as mild, moderate or severe depending on the presence of limitation of elevation in adduction, presence of downshoot on adduction and presence of vertical deviation in primary gaze with or without abnormal head posture (AHP) (Stager et al. 1999). Acquired cases have been reported in rheumatoid arthritis (Cooper et al. 1990) and sinusitis in the trochlear region (Brown 1973), or following inferior temporal orbital scarring (iatrogenic Brown syndrome), whereas congenital Brown syndrome is attributed to inelasticity of the superior oblique (SO) (Parks and Brown 1975; Wright 1999). Brown syndrome can also be simulated in cases of pulley instability and inferior displacement of the lateral rectus muscle (Wright 1999). All aetiologies of Brown syndrome will have a positive FDT, a diagnostic factor which aids in the differential diagnosis from inferior oblique (IO) paresis (Ansons and Davis 2014). Other conditions from which Brown syndrome should be differentiated include double elevator palsy, Graves’ Orbitopathy, orbital floor blowout fracture, congenital fibrosis of the extraocular muscles, myasthenia gravis and multiple sclerosis (Cooper et al. 1990).

Brown syndrome is commonly reported as a stable condition or one that improves over time (Ansons and Davis 2014). Kaban et al. (1993) and Dawson et al. (2008) identified between 10% (6/60) to 75% (24/32) of congenital Brown cases show an improvement in ocular movements over time. Wright (1999) identified spontaneous improvement in 16% (5/32) of acquired Brown syndrome patients compared to congenital Brown syndrome patients, with a smaller number of congenital cases undergoing surgery compared to acquired cases.

Surgical intervention is indicated in Brown syndrome when a large hypotropia, AHP, diplopia or the potential to establish/regain fusion is present (Ansons and Davis 2014). Traditionally the SO is weakened by tenotomy/tenectomy (Ansons and Davis 2014), recession (Ansons and Davis 2014) or by the use of a tendon expander (Stager et al. 1999; Wright 1999; Pollard and Greenberg 1999) or a suture bridge (Suh et al. 2008). Of 15 Brown syndrome patients who underwent SO tendon expander surgery, Wright (1999) reported 87% (13/15) were corrected with a single surgery, 14 showed improved motility and 10 showed normal versions after surgery. Keskinbora (2007) reported all eyes (22 eyes, 16 patients) achieved elevation in adduction following SO tendon expander surgery and no cyclotorsion occurred.

A unique case of progressive unilateral Brown syndrome is described with documented clinical findings over 14 years, during which time the patient sought opinions on the management options for intermittent diplopia and increasing AHP. The current reported Brown syndrome case is atypical in presentation, variation, duration and progression with no underlying pathology/aetiology found to explain the diagnosis or the progression.

## Case report

### Presentation

**Figure 1:**
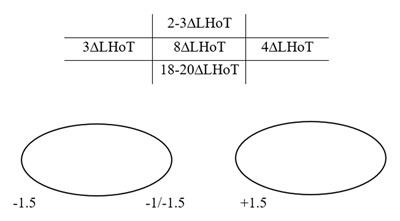
Initial presentation. Five positions of gaze PCT and ocular movements. LHoT: left hypotropia, Δ: prism dioptres.

A 28-year-old male presented complaining of intermittent diplopia. He had a slight head tilt left AHP. The cover test revealed an intermittently controlled left hypophoria at near and at distance, with worse control in the distance. Binocular single vision (BSV) was present at near, but convergence was reduced. Ocular movements and prism cover test (PCT) measurements are shown in Figure 1 (all PCT measurements are alternate PCT, in free space, at 6m fixation). No restriction in elevation in adduction was recorded. A qualitative judgement of positive Bielschowsky head tilt test (BHTT), tilting to the right, was recorded though no measurement was recorded. Potential diagnoses were right inferior rectus (IR) palsy, right SO palsy and left IO palsy. Fresnel prisms (4Δ base up left lens) to help control the primary position deviation were prescribed, as well as prism fusion range and convergence exercises. Later prisms (4Δ base up left lens) were incorporated into glasses.

### One year after presentation

Despite prism incorporation the patient returned one year later with gradually worsening diplopia symptoms keen to discuss surgical options. All clinical findings were similar and no AHP was observed. The patient declined surgery.

### Four years after presentation

**Figure 2:**
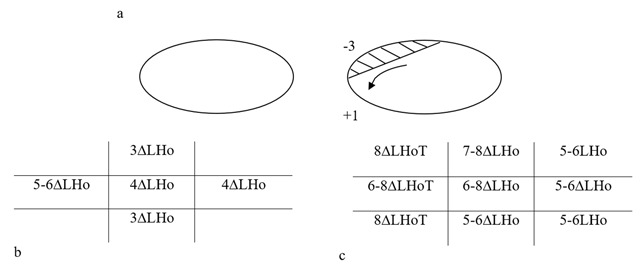
Ocular motility four years after presentation **a).** PCT measurements before **b)** and after **c)** four hours of monocular occlusion. LHo: left hypophoria.

The patient returned once again seeking a surgical opinion as the deviation was constantly manifest on right gaze with diplopia. Although no consistent AHP was adopted, he occasionally used a head tilt left AHP to aid control of his diplopia. The incorporated prisms were no longer helpful, so had been abandoned. Investigation of thyroid function and acetylcholine receptor antibodies gave normal results. A –3 underaction and limitation of the left eye elevating in adduction and a down drift of the left eye on dextroversion was recorded (Figure 2a). No click or underaction of the right IR or right SO was detected. The patient was diagnosed with a decompensating left Brown syndrome. The patient was re-measured following four hours of monocular occlusion, the deviation doubled in size as shown in Figure 2c compared to Figure 2b. Once again the patient declined surgery.

### Nine years after presentation

**Figure 3:**
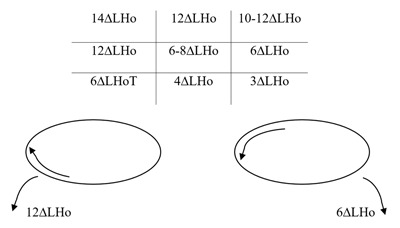
Nine years after presentation. Nine positions of gaze PCT, ocular movements and BHTT.

The patient returned complaining of worsening intermittent diplopia and using a larger AHP, 15° left head tilt (qualitative judgement), to maintain BSV. The deviation was unchanged in primary position compared to five years ago, yet had increased on right gaze (Figure 3). The differential diagnosis was a left IO palsy or a left atypical Brown syndrome. No torsion was measured in primary position, upgaze or downgaze (double Maddox rod) and no limitation of the LE was detected. The patient again declined surgery.

### Thirteen years after presentation

**Figure 4:**
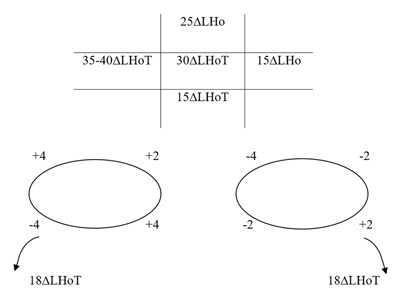
Thirteen years after presentation. Five positions of gaze PCT, ocular movements and BHTT.

The patient was again struggling to maintain BSV when tired despite the AHP (head tilt left) and increasingly complaining of blur. The cover test showed a moderate left hypotropia with diplopia, which was controlled to a hypophoria with the AHP. Ocular motility and negative BHTT findings are shown in Figure 4. Fundus examination revealed slight left incyclotropia. The patient decided to proceed with surgery to try and improve diplopia symptoms and reduce the AHP.

### Fourteen years after presentation – preoperative assessment (in clinic)

**Figure 5:**
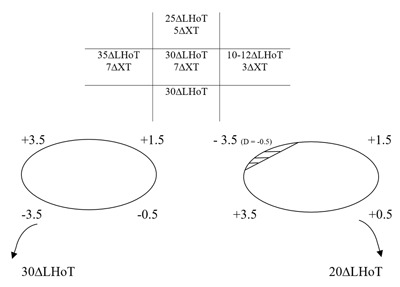
Preoperative assessment. Five positions of gaze PCT, ocular movements and BHTT. D: duction.

The ocular movements and PCT measurements are shown in Figure 5. Left incyclotorsion of 10° was measured in primary position at 1/3 m (double Maddox rod).

### Strabismus surgery procedure – 14 years after presentation

Intraoperative FDT demonstrated a –2 restriction of the left eye elevating in adduction only, confirming the diagnosis of left Brown syndrome. A 7 mm tendon spacer was inserted into the left SO tendon and a 3 mm recession of the right superior rectus (SR) was performed. FDT was normalised following spacer insertion.

### One day postoperative

The patient reported blur in the right eye, but no diplopia. Cover test revealed a minimal left hypotropia without diplopia, which was controlled to a phoria with a slight head tilt left AHP. Full extremes of gaze could not be tested, yet there was reversal of the vertical deviation on right and left gaze, with no diplopia.

### One month postoperative

**Figure 6:**
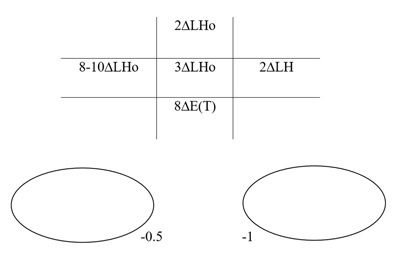
One month postoperative. Five positions of gaze PCT and ocular movements. LH: left hyperphoria, E(T): intermittent esotropia.

No AHP was evident and the patient’s diplopia symptoms were vastly improved. Cover test revealed a minimal left hypophoria with good recovery and BSV in primary position. Ocular movements and PCT measurements are shown in Figure 6. Diplopia was only reported on extremes of gaze, and the patient was happy with the postoperative results.

### One year postoperative

**Figure 7:**
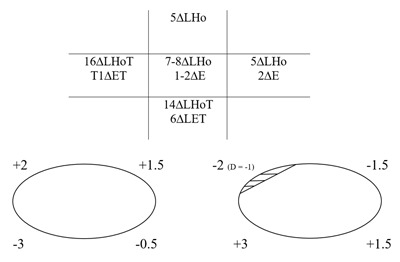
One year postoperative. Five positions of gaze PCT and ocular movements. E: esophoria, ET: esotropia.

A very slight tilt left AHP was evident and the patient reported occasional diplopia in elevation and depression. Cover test with AHP revealed a minimal left hypophoria at near with delayed recovery and minimal left hypophoria at distance with good recovery. Ocular movements and PCT measurements are shown in Figure 7. Without AHP the patient’s deviation and control were recorded as slightly worse. The patient still reports surgery has improved his diplopia and AHP.

## Discussion

During the 14 years prior to surgery, this patient had documented progression of his ocular motility findings, which were confirmed by FDT as left Brown syndrome. The previous differential diagnoses were left Brown syndrome, left IO palsy or possibly right IR palsy. IO and IR palsies are differentiated from Brown syndrome by the lack of limitation on ductions, ‘A’ pattern, positive BHTT, the presence of incyclotorsion and muscle sequelae (Ansons and Davis 2014; Manley and Rizwan 2011; Wright 1999; Scott and Nankin 1977; Greenberg and Pollard 2005). When a vertical deviation is due to paresis or overaction of a vertically acting muscle, this deviation is exaggerated by head tilting because of the stimulation of unequal pairs of vertically acting muscles (Hertle 1993). Brown syndrome may look like the IO is the primary affected muscle but it does not typically have increased vertical deviation on head tilt (Kaban et al. 1993). Our patient showed a positive BHTT at presentation, and this was measured nine years after presentation, suggesting an IO or IR paresis (Scott and Nankin 1977); however, the BHTT was at times negative over the follow-up period. No torsional deviation was measured initially, but left incyclotorsion was identified 13 years after presentation (fundus examination) and measured during preoperative assessment (10° double Maddox rod), suggesting a left IO paresis, rather than Brown syndrome. Donahue et al. (2001) compared IO paresis with vertical skew deviation and found all IO paresis patients had incyclotorsion of the hypotropic eye, which is in keeping with our patient. During the 14-year period before surgery, muscle sequelae development was more in keeping with left IO or right IR palsy (Ansons and Davis 2014), rather than the expected right SR overaction if it were Brown syndrome; however, the lack of horizontal deviation and presence of V pattern (Ansons and Davis 2014) (minimal in our patient) are more typical of Brown syndrome.

Brown syndrome is commonly reported as a stable condition, or one that improves over time (Cooper et al. 1990; Kaban et al. 1993; Dawson, Barry and Lee 2008). Progression or decompensation of Brown syndrome has been previously reported in 3.3% (2/60) (Kaban et al. 1993) and 9.6% (5/32) (Dawson, Barry and Lee 2008) of cases. Over 14 years our patient showed a gradual worsening of his diplopia and AHP, and ocular motility that at times looked like Brown syndrome, but at others less so. No acquired aetiology or anatomical anomaly was detected to explain the progressive Brown syndrome documented in our patient. If surgery had proven unsuccessful the clinical picture of an intermittent Brown syndrome may have been due to muscle pulley instability and inferior displacement of the lateral recti (Wright 1999).

Our patient eventually elected for strabismus surgery. Traditionally a SO weakening procedure is performed by tenotomy (Hertle 1993), tendon spacer (Pollard and Greenberg 1999) or suture spacer (Suh, Oystreck and Hunter 2008). SO tenotomy, due to the uncontrolled separation between the cut ends of the tendon, carries a risk of overcorrection (Oh et al. 2002). To allow a controlled weakening effect, Wright (1999) proposed the SO silicone tendon expander as a reversible SO weakening procedure, which preserves the mechanics of the tendon. A suture spacer technique has been proposed (Suh, Oystreck and Hunter 2008) as a technically easier procedure than Wright’s silicone tendon expander. However, sutures may provide a scaffolding to encourage cut end reattachment and adhesions (Suh, Oystreck and Hunter 2008). Using a SO tendon expander allows the surgical dose to be graded for the severity of the Brown syndrome. Wright (1999) reports an 87% (13/15) success rate in correcting Brown syndrome patients after one surgery, increasing to 93% (14/15) with two surgeries. Stager and colleagues (1999) used longer lengths of silicone tendon expander dependent on Brown syndrome severity, reporting an improvement in all patients.

A 7 mm tendon expander was inserted into the left SO tendon of our patient and the right SR was recessed by 3 mm with good ocular motility outcomes. One day postoperatively no significant restrictions were observed. One month postoperatively the patient had small bilateral SO underactions, a well controlled phoria and only occasional diplopia on extremes of gaze. One year postoperatively the deviation had increased slightly and a small underaction and restriction of the left eye elevating in adduction was evident. Despite this, the patient controls his residual hypophoria well with a minimal AHP and only experiences diplopia on extreme elevation and depression.

## Conclusion

This atypical case of slowly progressive (over 14 years) unilateral Brown syndrome was eventually confirmed by intraoperative FDT. No underlying pathology/aetiology was found to explain the diagnosis or the progression. To reduce the AHP and improve diplopia symptoms a 7 mm left SO tendon spacer and 3 mm right SR recession were performed. These procedures were successful in reducing the AHP and improving diplopia symptoms up to one year postoperatively.
